# Impact of heart failure and preoperative platelet count on the postoperative short‐term outcome in infective endocarditis patients

**DOI:** 10.1002/clc.24171

**Published:** 2023-10-10

**Authors:** Junjie Wang, Suiqing Huang, Jian Hou, Kangni Feng, Huawei Wu, Quan Liu, Zhuoming Zhou, Huayang Li, Li Luo, Liqun Shang, Guangxian Chen, Zhongkai Wu

**Affiliations:** ^1^ Department of Cardiac Surgery First Affiliated Hospital of Sun Yat‐sen University Guangzhou China; ^2^ Department of Epidemiology, Mailman School of Public Health Columbia University New York New York USA; ^3^ Department of Cardiac Surgery First Affiliated Hospital of xi'an jiaotong university Xi'an Shaanxi China; ^4^ Department of Cardiothoracic Surgery ICU, First Affiliated Hospital Sun Yat‐sen University Guangzhou China

**Keywords:** cardiac surgery, heart failure, infective endocarditis, platelets, risk factor analysis

## Abstract

**Background:**

Heart failure (HF) and platelet count are often considered risk factors for mortality in patients with infective endocarditis (IE); however, their effects on various complications have not been elucidated.

**Hypothesis:**

We speculated that HF and platelet count have significant impact on the short‐term outcomes of IE.

**Methods:**

This single‐center retrospective study analyzed data from 320 IE patients who underwent surgery. A multivariate Cox proportional hazards model was used to identify the risk factors for adverse outcomes. The effect of the platelet count on the prognosis of patients with HF was determined by subgroup analysis and Kaplan–Meier analysis.

**Results:**

The study population was divided into the HF group (*n* = 102) and the non‐HF group (*n* = 218). The median age of the total population was 44.5 years (31–56 years), of which 227 (70.94%) patients were male. The incidence rates of 1‐year all‐cause mortality, cardiac outcomes, and composite outcomes were respectively almost sixfold, fourfold, and threefold higher in the HF group than in the non‐HF group (all *p* < 0.001). In multivariate Cox regression analysis, HF was an independent risk factor for 1‐year all‐cause mortality, cardiac outcomes, cerebral outcomes, and composite outcomes. The Kaplan–Meier survival curves revealed that the patients with both HF and thrombocytopenia demonstrated the worst composite outcomes than the patients of the other groups (log‐rank *p* < 0.001). In the HF group, the platelet count was significantly associated with mortality and composite outcomes.

**Conclusions:**

HF and preoperative platelet count are significantly associated with 1‐year all‐cause mortality and adverse outcomes postoperatively in IE patients. Patients with HF and thrombocytopenia have the worst short‐term prognosis.

## Graphical abstract

In this study, we found a mutual promoting effect between heart failure (HF) and thrombocytopenia. Platelets had a greater impact on the prognosis of HF patients than of non‐HF patients. Event‐free survival in patients with both HF and thrombocytopenia was significantly shorter than that in other patients.

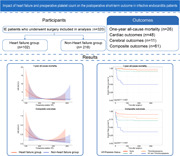

## 1 INTRODUCTION

Infective endocarditis (IE) is a systemic infectious disease characterized by valvular destruction and vegetation formation and is accompanied by serious complications and significant mortality.^1^, ^2^ Despite recent improvements in the diagnosis and antimicrobial and surgical management, the incidence of IE continues to increase every year.^3^

The condition of IE patients is complex and often accompanied by complications, such as acute renal failure, cardiovascular complications, neurological complications, and gastrointestinal complications, which are important causes of death and adverse outcomes.^4^ Although approximately half of IE patients receive surgical treatment and have a satisfactory overall therapeutic effect, the mortality rate remains as high as 8%.^5^, ^6^ Currently, several studies have identified some risk factors for mortality in IE patients, including heart failure (HF), platelet count, age, anemia, and bivalvular involvement.^7^, ^8^, ^9^ However, in addition to death, other systemic complications also deserve our attention. These complications not only increase the mortality risk but also reduce the quality of life and increase the patient's medical expenses and the length of hospital stay. For instance, cerebral infarction does not necessarily cause death; however, the associated hemiplegia and impaired consciousness greatly reduce the quality of life. Therefore, many details of the preoperative evaluation and management of IE remain to be optimized. In addition to assessing the mortality risk, timely intervention is crucial to reduce the incidence of complications.

There is an interrelationship between HF and platelets. On the one hand, HF affects platelet production, activation, and function.^10^ On the other hand, platelets interact with leukocytes and endothelial cells and release inflammatory factors that lead to the adhesion and migration of monocytes.^11^ Activated platelets may lead to disease progression in HF and the development of coronary atherosclerosis and microthrombosis, thereby increasing the risk of sudden death.^12^ A reduction in platelet count is independently associated with mortality and reduced ejection fraction in HF.^13^ IE has multiple pathophysiological processes, such as severe inflammatory response, HF, and thrombosis. Moreover, the pathophysiological mechanism of the vicious cycle between HF and thrombocytopenia is likely to affect the prognosis of IE; however, studies in this area are lacking.

This study aimed to investigate the risk factors for postoperative short‐term mortality and complications in patients with IE and to evaluate the effect of HF and platelet count on the prognosis of IE. Furthermore, we analyzed and discussed the influence of HF and platelets on various complications in IE patients.

## 2 METHODS

### 2.1 Study population and data collection

**Figure 1:**
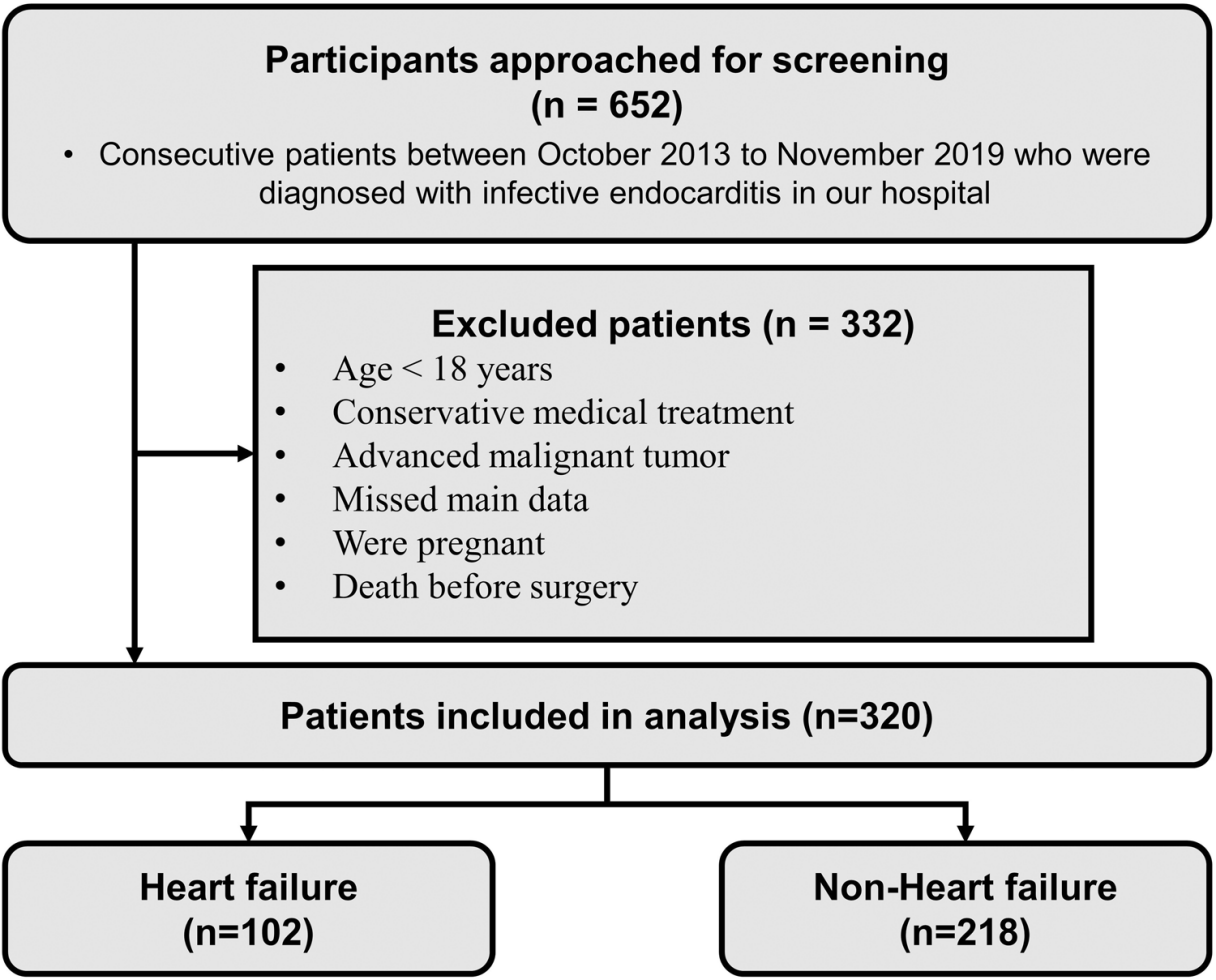
Flow chart for inclusion, exclusion, and grouping of patients with infective endocarditis. IE, infective endocarditis.

This retrospective observational study enrolled 652 consecutive patients diagnosed with IE at The First Affiliated Hospital of Sun Yat‐sen University between October 2013 and November 2019. All patients definitively fulfilled the modified Duke criteria for IE. The exclusion criteria were as follows: received conservative medical treatment (*n* = 278), missing data regarding the main variable (*n* = 13), death before surgery (*n* = 4), age < 18 years (*n* = 7), pregnancy (*n* = 2), advanced malignant tumors (*n* = 23), and participation in other clinical trials (*n* = 5). The main analysis included 320 patients who underwent cardiac surgery for IE (Figure 1). According to the European Society of Cardiology Guidelines for the management of IE, all patients' surgical indications and medical management were evaluated by the endocarditis team.^4^ In total, 50 potential variables were collected and analyzed separately, including demographics, medical history, clinical characteristics, laboratory results, echocardiography findings, and surgical details. This study was reviewed and approved by the Institutional Review Board of The First Affiliated Hospital of Sun Yat‐sen University (approval number: 2018 [100]) and was consistent with the Declaration of Helsinki. The authors confirm that patient consent is not applicable to this article because it is a retrospective case report using deidentified data.

### 2.2 Definition and outcomes

All investigators were blinded to the patient information. The preoperative platelet count was obtained from the last routine blood test before surgery, usually within 3 days. Thrombocytopenia, a categorical variable, was defined as platelet count < 150 × 10^9^/L.^14^ The minimum inpatient value of serum creatinine during the first 7 days of admission was used as the baseline creatinine level. Preoperative renal insufficiency was defined as acute kidney injury or chronic kidney disease stages 3–5 according to the Kidney Disease Improving Global Outcomes criteria.^15^, ^16^ The postoperative adverse outcomes were classified as follows: (1) all‐cause mortality occurring during hospitalization or within 1 year postoperatively; (2) cardiac outcomes, including acute HF, acute myocardial infarction, cardiac arrest requiring cardiopulmonary resuscitation, malignant arrhythmia, shock, acute pericardial tamponade, or use of ventricular assist devices such as extracorporeal membrane oxygenation (ECMO) or intra‐aortic balloon pump; (3) cerebral outcomes, including cerebral hemorrhage, cerebral infarction and cerebral hernia confirmed on computed tomography or magnetic resonance imaging, or transient ischemic attack; and (4) composite outcomes, including gastrointestinal bleeding or ischemia, large retroperitoneal hematoma, pulmonary embolism, septic shock, and other conditions that caused significant symptoms or were life‐threatening, in addition to the aforementioned adverse outcomes. When more than two adverse events occurred, the earliest time was recorded as the composite adverse event time. All postoperative complications were confirmed by experienced specialists. Comorbidities were defined according to the International Classification of Diseases, Ninth Revision, Clinical Modification.

### 2.3 Statistical analysis

Continuous variables are presented as means ± standard deviations or as medians with interquartile ranges based on normality. Categorical variables are presented as group frequencies and percentages. The student's *t*‐test and Mann–Whitney *U*‐test were used to compare continuous variables. The *χ* ^2^ test or Fisher's exact test was used to compare categorical variables. Subgroup and multivariate analyses of the association between covariates and adverse outcomes were performed using a Cox proportional hazards model. The assumption of proportional hazards was tested using scaled Schoenfeld residuals. Based on previous research and medical experience, the following covariates were considered to have a potential influence on the adverse outcomes: age, HF, double‐valve IE, right‐sided IE, atrial fibrillation, vegetation size > 10 mm, and preoperative hemoglobin levels. Kaplan–Meier analysis was conducted to estimate the event‐free survival of adverse outcomes, with log‐rank tests used to discriminate between the Kaplan–Meier curves. The visualized relationship between the preoperative platelet count and adverse outcomes in different HF subgroups was assessed using restricted cubic spline (RCS) curves. All tests were two‐tailed, and *p* < .05 were considered statistically significant. All statistical analyses were performed using the SPSS (version 26.0) software (SPSS Inc.) and the R statistical package (The R Foundation; http://www.r-project.org; version 4.1.3).

## 3 RESULTS

### 3.1 Baseline characteristics

This study included 320 hospitalized IE patients who had undergone cardiac surgery from October 2013 to November 2019. The study population was divided into the HF group (31.88%, *n* = 102) and the non‐HF group (68.12%, *n* = 218). The demographic characteristics and medical data of the participants are summarized in Table 1.

**Table 1:** Clinical characteristics and outcomes of IE patients undergoing surgery with and without heart failure.

Characteristics	Total (*n* = 320)	HF (*n* = 102)	Non‐HF (*n* = 218)	*p* Value
Age, years	44.5 (31−56)	49.5 (36−61)	41.5 (29−54)	**.002** a
Gender, male	227 (70.94)	75 (73.53)	152 (69.72)	.485
Smoking	47 (14.69)	20 (19.61)	27 (12.39)	.089
*Comorbidities*				
Diabetes mellitus	14 (4.38)	6 (5.88)	8 (3.67)	.367
Hypertension	53 (16.56)	25 (24.51)	28 (12.84)	**.009** a
COPD	4 (1.25)	3 (2.94)	1 (0.46)	.063
Preoperative renal failure	75 (23.44)	44 (43.14)	31 (14.22)	**<.001** a
Congenital heart disease	57 (17.81)	10 (9.8)	47 (6.52)	**.01** a
Coronary heart disease	15 (4.69)	7 (6.86)	8 (3.67)	.208
Atrial fibrillation	27 (8.44)	10 (9.8)	17 (7.8)	.547
Previous cardiac surgery	21 (6.56)	5 (4.9)	16 (7.34)	.412
Peripheral vascular disease	9 (2.81)	1 (0.98)	8 (3.67)	.175
Preoperative stroke	68 (21.25)	23 (22.55)	45 (20.64)	.698
*Echocardiography*				
Infected valve				
Aortic	156 (48.75)	63 (61.76)	93 (42.66)	**.001** a
Mitral	182 (56.88)	55 (53.92)	127 (58.26)	.466
Double valve	51 (15.94)	23 (22.55)	28 (12.84)	**.027** a
Posthetic valve	13 (4.06)	4 (3.92)	9 (4.13)	.93
Right‐Sided IE	27 (8.44)	7 (6.86)	20 (9.17)	.488
Vegetation size, mm	11 (6.2−16)	11.8 (7−15.4)	11 (6−16)	.626
*Perivalvular complications*				
Valvular perforation	101 (31.56)	45 (44.12)	56 (25.69)	**.001** a
Chordae tendineae rupture	49 (15.31)	17 (16.67)	32 (14.68)	.645
Perivalvular abscess	30 (9.38)	14 (13.73)	16 (7.34)	.068
LVEF, %	67.5 (62−73)	67 (60−73)	68 (62−73)	.519
Pulmonary artery systolic pressure ≥ 40 mmHg	122 (38.13)	48 (47.06)	74 (33.94)	**.024** a
*Microbiological data*				
*Staphylococcus aureus*	23/274 (8.39)	12/89 (13.48)	11/185 (5.95)	**.035** a
*Streptococcus spp.*	67/274 (24.45)	19/89 (21.35)	48/185 (25.95)	.407
*Enterococcus spp.*	6/274 (2.19)	3/89 (3.37)	3/185 (1.62)	.354
Gram‐negative bacteria	15/274 (5.47)	6/89 (6.74)	9/185 (4.86)	.522
Gram‐positive bacteria	147/274 (53.65)	44/89 (49.44)	103/185 (55.68)	.332
Negative blood cultures	111/274 (40.51)	39/89 (43.82)	72/185 (38.92)	.439
Fungi	1/274 (0.36)	0 (0.00)	1/185 (0.54)	.487
*Laboratory tests results*				
White blood cell, 10^9^/L	8.21 (6.43−10.81)	9.02 (7.18−11.67)	7.82 (6.24−10.31)	**.005** a
Hemoglobin, g/L	110 (94.25−128)	104 (86−126)	115 (97−129)	**.002** a
Hematocrit, %	33.5 (29.23−38.88)	31.6 (26.3−38.35)	34.55 (30.08−39.1)	**.006** a
Platelets, 10^9^/L	246.73 ± 93.10	230.75 ± 92.14	254.21 ± 92.81	**.035** a
Creatinine, umol/L	75 (61−91)	83 (70−113.25)	71 (58−86)	**<.001** a
Urea nitrogen, mmol/L	5.4 (4.2−7.3)	6.95 (5.08−10.23)	5 (4−6.33)	**<.001** a
Serum albumin, g/L	35.99 ± 5.39	34.8 ± 5.34	36.54 ± 5.34	**.007** a
AST, U/L	23 (18−31)	25 (18.75−36.18)	22 (18−30)	**.01** a
Total bilirubin, umol/L	12.65 (9.33−16.68)	13.45 (9.98−17.73)	12.3 (9−16.23)	.112
*Outcome events*				
Composite outcomes	61 (19.06)	37 (34.26)	24 (11.01)	**<.001** a
1‐year all‐cause mortality	26 (8.13)	19 (18.63)	7 (3.21)	**<.001** a
Cardiac outcomes	48 (15)	31 (28.70)	17 (7.80)	**<.001** a
Arrhythmia	19 (5.94)	10 (9.26)	9 (4.13)	**.045** a
Acute heart failure	21 (6.56)	13 (12.04)	8 (3.67)	**.002** a
Shock	22 (6.88)	17 (15.74)	5 (2.29)	**<.001** a
Cerebral outcomes	11 (3.44)	7 (6.48)	4 (1.83)	**.021** a
Cerebral infarction	3 (0.94)	2 (1.85)	1 (0.46)	.194
Cerebral hemorrhage	6 (1.88)	4 (3.70)	2 (0.92)	.065

*Note*: Bold values indicate statistical differences in variables between the heart failure group and the non‐heart failure group (*p* < .05). Continuous variables are presented as means ± standard deviations or as medians with interquartile ranges based on normality. Categorical variables are presented as group frequencies and percentages.

Abbreviations: AST, aspartate transaminase; COPD, chronic obstructive pulmonary disease; HF, heart failure; LVEF, left ventricular ejection fraction.

^a^ Statistically significant.

The median age of the total population was 44.5 years (31–56 years), of which 227 (70.94%) patients were male. The most common valve vegetations were mitral valve vegetations (*n* = 182, 56.88%), and approximately one in six patients (*n* = 51, 15.94%) had a double‐valve infection. Furthermore, the most common perivalvular complication was valvular perforation (*n* = 101, 31.56%), and 83 patients (25.94%) underwent urgent surgery. The most common surgical indication was uncontrolled infection, accounting for 75.63%. Surgical indications were not significantly different between the two groups (all *p* > .05), except the indication of HF (Table 2). The mean preoperative platelet count was 246.73 ± 93.1 × 10^9^/L. Approximately 15.62% of patients (*n* = 50) had a platelet count < 150 × 10^9^/L, while 3.75% (*n* = 12) had a platelet count < 100 × 10^9^/L. Pathogenic microorganisms were identified in 274 cases (85.63%), and the most common bacteria was *Streptococcus spp*. detected in 67 patients (24.45%).

**Table 2:** Surgical indications and details of IE patients with and without heart failure.

	Total (*n* = 320)	HF (n = 102)	Non‐HF (*n* = 218)	*p* Value
*Surgery indications*				
Heart failure	102 (31.88)	102 (100)	0	**<.001** a
Severe valve regurgitation	209 (65.31)	74 (72.55)	135 (61.93)	.063
Uncontrolled infection	242 (75.63)	81 (79.41)	161 (73.85)	.280
Prevention of embolism	166 (51.88)	52 (50.98)	114 (52.29)	.827
*Surgery details*				
Operative date, day	10 (7−16)	11 (7−18)	10 (7‐15)	.781
Urgent surgery	83 (25.94)	28 (27.45)	55 (25.23)	.673
Elective surgery	143 (44.69)	41 (40.2)	102 (46.79)	.269
Valve replace				
Aortic	156 (48.75)	65 (63.73)	91 (41.74)	**<.001** a
Mitral	194 (60.63)	58 (56.86)	136 (62.39)	.346
Tricuspid	18 (5.63)	5 (4.9)	13 (5.96)	.701
Pulmonary	4 (1.25)	0	4 (3.92)	.169
Valve replace				
Bioprosthetic	73 (22.81)	29 (28.43)	44 (20.18)	.101
Mechanical	231 (72.19)	69 (67.65)	162 (74.31)	.215

*Note*: Categorical variables are presented as group frequencies and percentages.

^a^ Statistically significant.

The incidence of HF was 31.88% (*n* = 102). Compared with the non‐HF group patients, those in the HF group were older and had more comorbidities such as hypertension, preoperative renal failure, congenital heart disease, and pulmonary hypertension, higher incidence of double‐valve infection, higher frequency of positive blood cultures for *Staphylococcus aureus*, and higher levels of white blood cells, creatinine, urea nitrogen, aspartate aminotransferase, and total bilirubin. However, the hemoglobin, platelets, and serum albumin were lower in the HF group than in the non‐HF group (all *p* < .05).

### 3.2 Postoperative outcome and Cox regression analysis

During the 1‐year follow‐up period, composite adverse outcomes were reported in 19.06% of the patients (*n* = 61). Additionally, 26 patients (8.13%) died, 48 (15%) had cardiac outcomes, 11 (3.44%) had cerebral outcomes, and 26 (8.13%) had two or more adverse events (Table 1). Notably, the incidence of adverse events was significantly higher in the HF group than in the non‐HF group. The incidence rates of 1‐year all‐cause mortality, cardiac outcomes, and composite outcomes were respectively almost sixfold, fourfold, and threefold higher in the HF group than in the non‐HF group (all *p* < .001).

Table 3 shows the multivariate Cox regression analysis of the four major postoperative adverse outcomes. After adjustment for age, double‐valve IE, right‐sided IE, atrial fibrillation, vegetation size > 10 mm, and preoperative hemoglobin levels, HF was considered an independent risk factor for 1‐year all‐cause mortality (adjusted hazard ratio [HR] = 5.050; 95% CI, 2.067–12.340; *p* < .001), cardiac outcomes (adjusted HR = 3.556; 95% CI, 1.937–6.527; *p* < .001), cerebral outcomes (adjusted HR = 3.671; 95% CI, 1.059–12.730; *p* = .04), and composite outcomes (adjusted HR = 3.086; 95% CI, 1.796–5.303; *p* < .001). Moreover, the preoperative platelet count was significantly associated with an increased risk of 1‐year all‐cause mortality (adjusted HR = 0.989; 95% CI, 0.983–0.995; *p* < .001), cardiac outcomes (adjusted HR = 0.996; 95% CI, 0.993–1; *p* = .049), and composite outcomes (adjusted HR = 0.996; 95% CI, 0.992–0.999; *p* = .007). Thus, both HF and preoperative platelet count were considered independent risk factors for poor prognosis.

**Table 3:** Multivariable cox regression analysis of adverse outcomes.

Risk factors	Total		HF group		Non‐HF group	
	Hazard ratio (95% CI)	*p* Value	Hazard ratio (95% CI)	*p* Value	Hazard ratio (95% CI)	*p* Value
*1‐years mortality*						
Age, years	1.000 (0.977−1.024)	.981	0.984 (0.958−1.010)	.232	1.078 (1.009−1.150)	**.025** a
Heart failure	5.050 (2.067−12.340)	**<.001** a	NA	NA	NA	NA
Double valve IE	4.432 (1.887−10.405)	**.001** a	5.930 (2.237−15.723)	**<.001** a	1.611 (0.166−15.586)	.681
Right‐Sided IE	3.746 (1.458−9.626)	**.006** a	2.013 (0.624−6.489)	.241	15.126 (2.645−86.513)	**.002** a
Preoperative Platelets, 10^9^/L	0.989 (0.983−0.995)	**<.001** a	0.984 (0.976−0.991)	**<.001** a	0.998 (0.989−1.008)	.727
*Cardiac outcomes*						
Age, years	1.017 (0.998−1.037)	.075	1.008 (0.985−1.031)	.499	1.041 (1.008−1.076)	**.016** a
Atrial fibrillation	1.362 (0.593−3.129)	.466	2.083 (0.817−5.307)	.124	0.408 (0.052−3.187)	.393
Heart failure	3.556 (1.937−6.527)	**<.001** a	NA	NA	NA	NA
Double valve IE	1.594 (0.825−3.081)	.165	2.372 (1.121−5.016)	**.024** a	0.340 (0.044−2.611)	.214
Vegetation size >10 mm	1.166 (0.655−2.074)	.601	1.569 (0.750−3.283)	.232	0.835 (0.320−2.176)	.712
Preoperative Platelets, 10^9^/L	0.996 (0.993−1.000)	**.049** a	0.996 (0.992−1.001)	.115	0.996 (0.991−1.002)	.214
*Cerebral outcomes*						
Age, years	0.967 (0.930−1.006)	.099	0.964 (0.918−1.013)	.152	0.971 (0.907−1.040)	.405
Atrial fibrillation	3.186 (0.597−17)	.175	2.202 (0.216−22.494)	.506	6.517 (0.502−84.607)	.152
Heart failure	3.671 (1.059−12.730)	**.040** a	NA	NA	NA	NA
Preoperative Platelets, 10^9^/L	0.997 (0.990−1.004)	.386	0.993 (0.984−1.003)	.152	1.004 (0.992−1.016)	.532
Bioprosthetic valve replace	5.212 (1.461−18.595)	**.011** a	5.492 (1.002‐30.114)	**.050** a	6.344 (0.761‐52.882)	.088
*Composite outcomes*						
Age, years	1.014 (0.998−1.031)	.086	1.006 (0.986−1.027)	.565	1.030 (1.002−1.059)	**.038** a
Heart failure	3.086 (1.796−5.303)	**<.001** a	NA	NA	NA	NA
Double valve IE	1.592 (0.864−2.932)	.136	2.275 (1.124−4.604)	**.022** a	0.613 (0.140−2.682)	.516
Right‐Sided IE	2.225 (1.073−4.615)	**.032** a	1.357 (0.440−4.189)	.595	3.474 (1.320−9.145)	**.012** a
Preoperative Hemoglobin, g/L	0.997 (0.987−1.007)	.529	1.000 (0.987−1.013)	.948	0.990 (0.973−1.008)	.294
Preoperative Platelets, 10^9^/L	0.996 (0.992−0.999)	**.007** a	0.994 (0.990−0.999)	**.011** a	0.996 (0.992−1.001)	.117

Abbreviations: CI, confidence interval; HF, heart failure; NA, not applicable; OR, odds ratio.

^a^ Statistically significant.

### 3.3 Subgroup analysis

Subgroup multivariate Cox regression analysis was performed to explore the interaction between HF and preoperative platelet count further (Table 3). In the HF group, the preoperative platelet count continued to be associated with 1‐year all‐cause mortality (adjusted HR = 0.984; 95% CI, 0.976–0.991; *p* < .001) and composite outcomes (adjusted HR = 0.994; 95% CI, 0.990–0.999; *p* = .011). However, in the non‐HF group, the platelet count was not an independent risk factor for any outcome (all *p* > .05).

**Figure 2:**
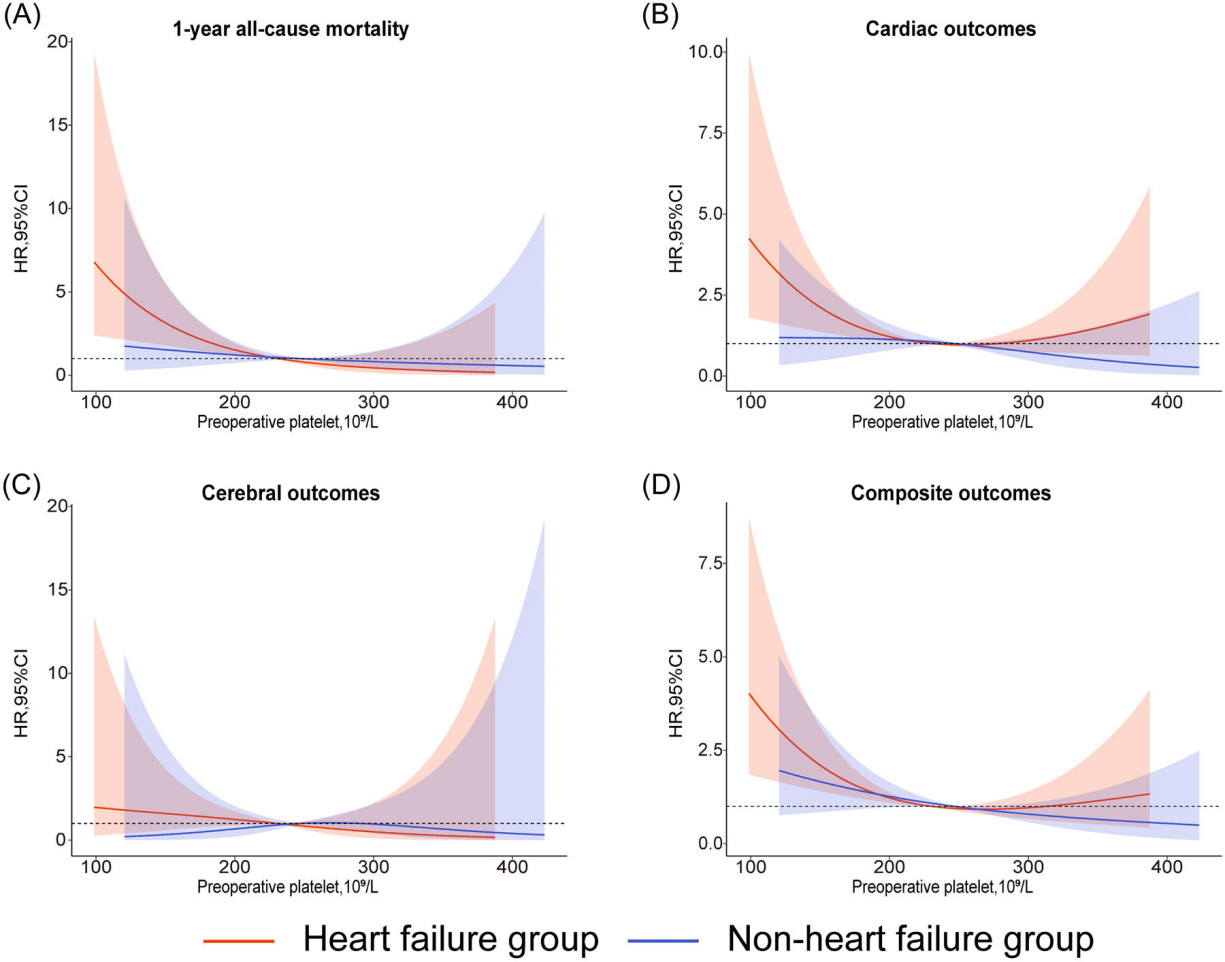
The restricted cubic spline analysis on the association between the preoperative platelet counts and the risk of adverse outcomes in different heart failure subgroups (A) 1‐year all‐cause mortality (B) Cardiac outcomes (C) Cerebral outcomes (D) Composite outcomes. CI, confidence interval; HR, hazard ratio.

Based on the stratification by HF, the relationship between the preoperative platelet count and the risk of various outcomes was analyzed using RCS. Using the cardiac outcomes (Figure 2B) and composite outcomes (Figure 2D) as the end events, a U‐shaped association was observed between the preoperative platelet count and risk in the HF group, with a higher risk than that in the non‐HF group. However, the preoperative platelet count was linearly associated with cardiac outcomes and composite outcomes in the non‐HF group (Figures 2B,2D). Using 1‐year all‐cause mortality (Figure 2A) and cerebral outcomes (Figure 2C) as the end events, an L‐shaped association was observed between the preoperative platelet count and risk in the HF group, with a higher risk than that in the non‐HF group only when the platelet count was low.

**Figure 3:**
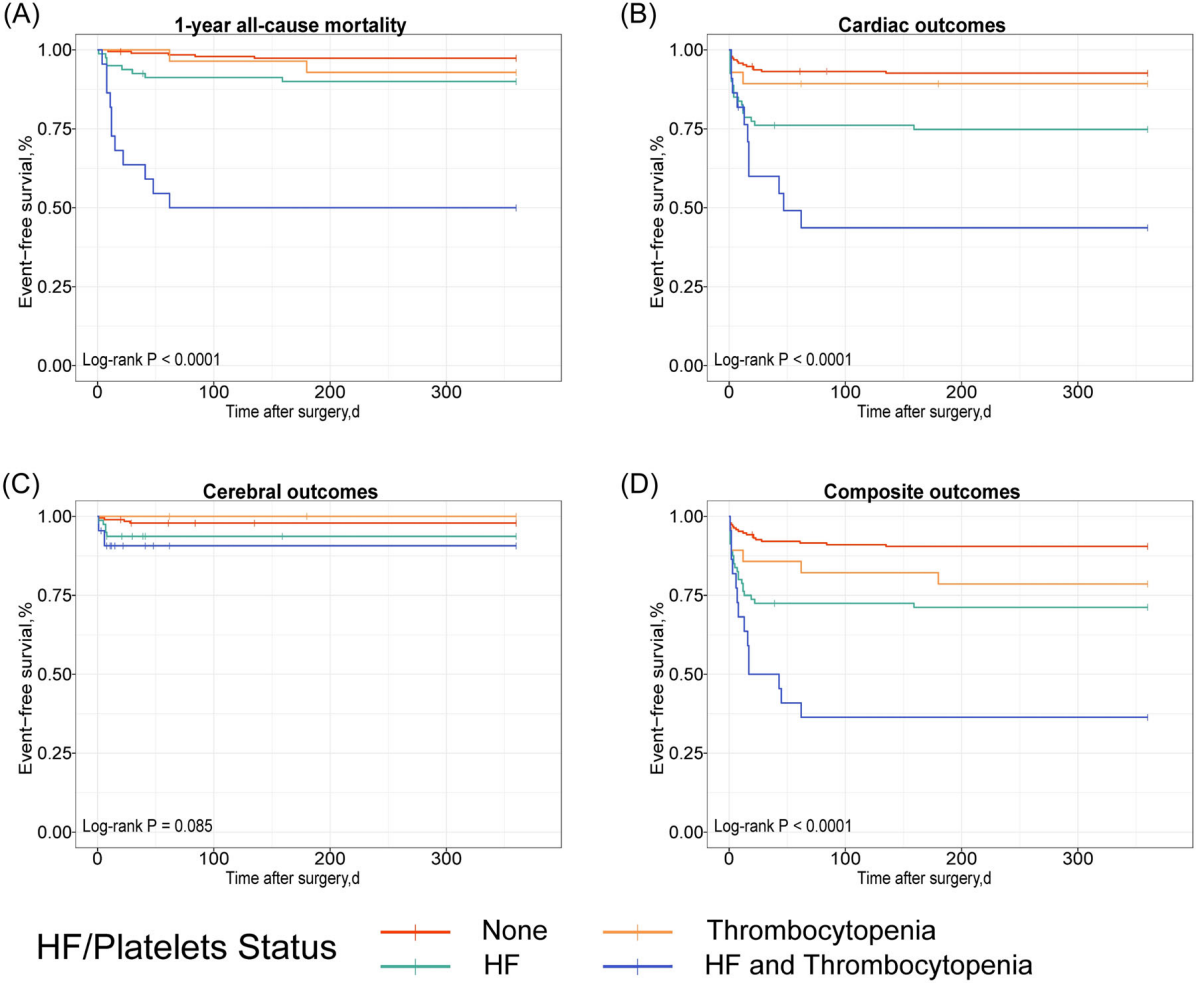
The Kaplan–Meier survival curves estimates of event‐free survival for adverse outcomes associated with thrombocytopenia and heart failure (A) 1‐year all‐cause mortality (B) Cardiac outcomes (C) Cerebral outcomes (D) Composite outcomes.

We divided the study sample according to the preoperative platelet count into two groups as follows: no thrombocytopenia (>150 × 10^9^/L; *n* = 270, 84.38%) and thrombocytopenia (<150 × 10^9^/L; *n* = 50, 15.62%). The Kaplan–Meier curves revealed the relationship between adverse outcomes and HF and thrombocytopenia. Notably, the patients with both HF and thrombocytopenia demonstrated higher incidence rates of 1‐year all‐cause mortality (Figure 3A), cardiac outcomes (Figure 3B), and composite outcomes (Figure 3D) than those of patients with either HF or thrombocytopenia or those with neither HF nor thrombocytopenia (all log‐rank *p* < .001).

## 4 DISCUSSION

This single‐center retrospective study evaluated the influence and interaction of HF and preoperative platelet count on short‐term postoperative outcomes in IE patients. We found that HF was significantly associated with the incidence of all postoperative adverse events, and preoperative platelet count was associated with 1‐year all‐cause mortality, cardiac outcomes, and composite outcomes. In the HF subgroup analysis, the preoperative platelet count retained its effect on 1‐year all‐cause mortality and composite outcomes. More importantly, the adverse effects of thrombocytopenia were amplified by HF. In the Kaplan–Meier survival analysis, patients with HF and thrombocytopenia had the worst short‐term prognosis.

Bacteremia and valve vegetation are important parts of the pathophysiology of IE. This vegetation is an excellent shelter for bacteria, which not only makes anti‐infective processes difficult but is also one of the main reasons for embolism and hemorrhage.^17^ Platelets, as the main component of vegetation, have been considered a promising target for IE management.^18^, ^19^ The application of antiplatelet therapy to reduce vegetation and emboli remains controversial in the clinical treatment of IE. In the management of platelets, there may be a transitional zone between promoting vegetation formation and increasing bleeding risk and controlling this balance is difficult. Some investigators have reported that antiplatelet treatment has a limited effect in reducing the risk of embolic events and is probably associated with an increased risk of bleeding.^20^, ^21^ Bleeding complications often lead to a worse prognosis in IE patients. Additionally, the platelet count was a significant predictor of early mortality after surgery in IE patients in other studies.^7^ Therefore, we should be alert regarding the risk of bleeding complications and death caused by a low platelet count, rather than vegetation formation and embolic events, especially under appropriate antibiotic therapy. Antimicrobial treatment is considered the best method to reduce the risk of embolic events and improve the prognosis of IE.^22^

IE patients encounter the double threat of pathogenic microorganisms and surgical trauma. Their baseline condition is poor, making them more vulnerable to HF and death. The role of platelets in the prognosis of IE has not yet been characterized, especially with respect to surgical trauma and severe infection. In this study of postoperative complications of IE, platelet count correlated significantly with cardiac outcomes and composite outcomes in addition to the 1‐year all‐cause mortality. This suggests that platelets may have a potential role in infection control, myocardial ischemia‐reperfusion injury, and organ protection other than thrombosis and bleeding. During sepsis, activated platelets have been shown to limit both bacterial growth and dissemination, and reduce bacterial load.^23^, ^24^ Cardiac outcomes were the primary postoperative adverse outcomes, accounting for 78.7% of the composite outcomes. Whether platelets can enhance myocardial protection is an important issue for discussion. It has been reported that platelet‐derived sphingosine‐1 phosphate can induce myocardial protection, and attenuate ischemia‐reperfusion injury.^25^ In a rat model of myocardial infarction, platelet gel was shown to preserve cardiac function and attenuate adverse ventricular remodeling without exacerbating cardiac inflammation.^26^ Additionally, activated platelets play a beneficial role in ischemia‐reperfusion injury.^27^ Although the aforementioned studies were not based on IE, therapies targeting platelets may be a unique and promising therapeutic approach to improve its prognosis.

HF is the most common complication of IE as well as the most common surgical indication for IE.^1^, ^4^ In our study, HF was present in almost one‐third of the patients, which concurred with previous studies.^28^ Currently, studies on IE complicated by HF focus on mortality. In addition to mortality, adverse events such as low cardiac output syndrome, malignant arrhythmias, bleeding events, or cerebral infarction deserve our attention. These complications can prolong the patient's hospital stay, increase medical costs, and reduce the quality of life. In our study, the patients with HF were associated with a significantly higher risk of 1‐year all‐cause mortality and adverse outcomes when compared with those without HF (all *p* < .05). The heart is the power source of systemic blood circulation. Hemodynamic disorder and organ hypoperfusion caused by HF are important causes of death and multiple organ dysfunction. First, although surgery is an important treatment to improve the prognosis of IE patients, cardiopulmonary bypass and cardiac arrest during surgery can cause serious trauma to the heart, especially in patients with preoperative HF symptoms. Most patients with HF have improved cardiac function and vegetation clearance postoperatively. However, a small part of patients develop low cardiac output syndrome and cardiogenic shock postoperatively and require ECMO to maintain circulation stability, which is associated with high mortality and complication rates.^29^ Second, pulmonary circulation is obstructed in patients with HF, leading to an accumulation of alveolar fluid that not only impairs bacterial clearance but also undermines the local defenses against infection, thereby increasing the risk of pneumonia.^30^, ^31^ The risk of cardiovascular and all‐cause death is overall three to fourfold higher after the onset of pneumonia, particularly in patients with valvular heart disease.^30^, ^31^, ^32^ Finally, the incidence of bivalvular involvement was higher in the HF group than in the non‐HF group (22.55% vs. 12.84%; *p* = .027) in our study, which meant longer cardiopulmonary bypass time and more cardiac structural damage, resulting in an increased incidence of embolic events and gastrointestinal complications.^8^, ^33^, ^34^

In addition to exploring the impact of HF and platelet count on the short‐term prognosis of IE patients, we found a mutual promoting effect between HF and thrombocytopenia. According to our findings, platelets had a greater impact on the prognosis of HF patients than of non‐HF patients. Event‐free survival in patients with both HF and thrombocytopenia was significantly shorter than that in other patients. Although we have reported a novel association between thrombocytopenia and HF, we acknowledge that the underlying mechanism remains uncertain, especially in the context of coagulopathy, inflammatory storm, and surgical trauma. In these patients, it is necessary to improve cardiac function and correct thrombocytopenia, which may lead to a better prognosis.

## 5 LIMITATIONS

Our study has several limitations. First, since this is a single‐center retrospective study, the generalizability of its conclusions needs further validation. However, the frequency of HF, death, and adverse outcomes in our study was similar to that reported in other studies. Second, all study data were obtained from electronic medical record systems and patient follow‐up, rather than prospective collection, which have inherent limitations. Several patients were excluded due to missing data, which may have introduced a potential bias. Finally, the platelet count was obtained from the last blood routine results before surgery. Some patients with thrombocytopenia on admission may have recovered after undergoing blood transfusion or medical treatment, and these interventions could potentially affect the prognosis.

## 6 CONCLUSIONS

The results of this study suggest that HF and preoperative platelet count are significantly associated with 1‐year all‐cause mortality and adverse outcomes postoperatively in IE patients. Patients with HF and thrombocytopenia have the worst short‐term prognosis.

## CONFLICT OF INTEREST STATEMENT

The authors declare no conflict of interest.

## Data Availability

The data that support the findings of this study are available from the corresponding author upon reasonable request.
